# Meeting Report: “Metagenomics, Metadata and Meta-analysis” (M3) Special Interest Group at ISMB 2009

**DOI:** 10.4056/sigs.641096

**Published:** 2009-12-29

**Authors:** Dawn Field, Iddo Friedberg, Peter Sterk, Renzo Kottmann, Frank Oliver Glöckner, Lynette Hirschman, George M. Garrity, Guy Cochrane, John Wooley, Jack Gilbert

**Affiliations:** 1 NERC Center for Ecology and Hydrology, Oxford, UK; 2 Department of Microbiology, Miami University, Oxford ,OH, USA; 3 Wellcome Trust Sanger Institute, Wellcome Trust Genome Campus, Hinxton, Cambridge, UK; 4 Microbial Genomics Group, Max Planck Institute for Marine Microbiology & Jacobs University Bremen, Bremen, Germany; 5 Information Technology Center, The MITRE Corporation, Bedford, MA, USA; 6 Department of Microbiology and Molecular Genetics, Michigan State University, East Lansing, Michigan USA; 7 European Molecular Biology Laboratory (EMBL), European Bioinformatics Institute (EBI), Wellcome Trust Genome Campus, Hinxton, Cambridge, UK; 8 University of California San Diego, La Jolla, CA, USA; 9 Plymouth Marine Laboratory (PML), Plymouth, UK

## Abstract

This report summarizes the proceedings of the “Metagenomics, Metadata and Meta-analysis” (M3) Special Interest Group (SIG) meeting held at the Intelligent Systems for Molecular Biology 2009 conference. The Genomic Standards Consortium (GSC) hosted this meeting to explore the bottlenecks and emerging solutions for obtaining biological insights through large-scale comparative analysis of metagenomic datasets. The M3 SIG included 16 talks, half of which were selected from submitted abstracts, a poster session and a panel discussion involving members of the GSC Board. This report summarizes this one-day SIG, attempts to identify shared themes and recapitulates community recommendations for the future of this field. The GSC will also host an M3 workshop at the Pacific Symposium on Biocomputing (PSB) in January 2010. Further information about the GSC and its range of activities can be found at http://gensc.org/.

## Introduction

There are now thousands of genomes and metagenomes available for study (http://www.genomesonline.org/) [1]. Interest in improved sampling of diverse environments (e.g. ocean, soil, sediment, and a range of hosts) combined with advances in the development and application of ultra-high throughput sequence methodologies is set to vastly accelerate the pace at which new metagenomes are generated. For example, in 2007, the Global Ocean Survey published scientific analyses of 41 metagenomes, and as of November 2009, the submission of user-generated metagenomes to the public MG-RAST Annotation server surpassed 4,000. We have now entered an era of “mega-sequencing” projects that include funded projects like the *Genomic Encyclopedia of Bacteria and Archaea* (GEBA) project [2] and the Human Microbiome Project (HMP) [3], with many more visionary projects on the horizon.

While a genome represents the full genetic (DNA) complement of a single organism, metagenomes represent the DNA of an entire community of organisms. Metagenomes are partial samples of complex and largely unknown communities that can only be poorly assembled. Genome and metagenomes are now also being complemented with studies of metatranscriptomes (community transcript profiles) and metaproteomes (community protein profiles). The comparative studies of these datasets, including multi-omic data from the same community, bring with them the need for new computational approaches. These data hold the promise of unparalleled insights into fundamental questions across a range of fields including evolution, ecology, environment biology, health and medicine. Advances stem from improved understanding of the combinations, abundances and functions of the organisms in these communities and their genes and pathways. We are just starting to exploit these technologies to understand the microbial world and have only scratched the surface in terms of sampling natural microbial diversity in terms of space and time.

Because the pace of genomic and metagenomic sequencing projects [4] is increasing rapidly, and will only accelerate as the application of ultra-high-throughput methods becomes more widespread, the role of standards is becoming ever more vital to scientific progress and data sharing. The Genomic Standards Consortium (GSC) is an international working body with the mission of developing richer descriptions of our collection of genomes and metagenomes through the development of standards and tools for supporting compliance and exchange of contextual information [5].

This report summarizes the proceedings of the "Metagenomics, Metadata and Meta-analysis”(M3) Special Interest Group at ISMB 2009. Special Interest Group meetings at ISMB are a specific way to bring together computational researchers interested in a particular topic. In hosting a SIG meeting, the GSC hoped to engage the wider bioinformatics research community in thinking about standards. The idea to hold a SIG meeting emerged during discussions at the GSC 6 workshop and the proposal was largely developed at the GSC 7 workshop in San Diego [6]. It was named M3 to cover the important intersections between the ongoing explosion of data (Metagenomics) and the ever growing need to support richer stores of associated contextual data (Metadata) to improve our ability to interpret and compare findings across large collections of independent studies (Meta-analysis).

The M3 SIG meeting explored the latest concepts, algorithms, tools, informatics pipelines, databases and standards that are being developed to cope with the analysis of vast quantities of metagenomic data. The goal of the GSC was to attract experimentalists and computational researchers making best use of available contextual information (metadata). We solicited abstract submissions describing comparative (meta) genomic studies that demonstrate the power of using contextual data curated (e.g. habitat or host) and measured (e.g. geographic location, salinity, temperature, or pH) in comparative metagenomic studies of large numbers of samples. For example, a recently published seminal paper illustrates the power of this approach to elucidate the relationships between metabolic pathways and environmental parameters in microbial communities [7] by using the data and metadata from the landmark Global Ocean Survey (GOS) study [8]. Additionally, host-derived examples such as the Human Microbiome Project and the resulting data sets were encouraged at the M3 SIG as they will open enormous new possibilities for integration and analysis of metagenomic data sets in this context. Likewise, studies that described new approaches, tools, databases, standards, ontologies or substantial new sets of curated metadata that aid in the integration and inter-operability of disparate datasets were welcomed. We also aimed to attract research focused on capture and organization of metadata, for example through text mining and ontology development that enables new understanding of the interaction of organisms in their ecological context.

The agenda of the M3 SIG meeting was designed to cover the marriage of science and standards. Through a series of invited and contributed talks, a panel discussion, and flash talks associated with a poster session, the organizers aimed to highlight scientific advances in the field and identify core computational challenges facing the wider community. Building such community-driven consensus, in the form of standards that support and accelerate scientific discovery in biology, is of growing importance. This is especially true given the rapid growth of experimental data, most notably including both genomic and metagenomic sequences.

### Session I: Metagenomics

The first session was organized to set the stage for the SIG by highlighting the vast amount of data that is being generated now and in the future. The session was chaired by Jeroen Raes (University of Brussels) and featured an invited talk by Owen White (University of Maryland) on the vision for the Human Microbiome Project's Data Analysis and Curation Centre (DACC). This was followed by two contributed talks on "Environment-Dependent Protein Domains in the GOS Metagenome" by Ivaylo Kostadinov (Max Planck Institute for Marine Microbiology). Jack Gilbert (Plymouth Marine Laboratory) talked about molecular characterization of the longer-term monitoring site "L4" in the Western English Channel. All three talks highlighted the vast amounts of data that are being generated by the genomics and metagenomics communities and the importance of standardized metadata for the analysis of these datasets.

These talks were followed by a series of one-minute-lightning-talks from all of the poster presenters.

### Session II: Metadata

The second session was chaired by Frank Oliver Glöckner (Max Planck Institute for Marine Microbiology). An overview of the current community-led standards landscape was given by Susanna Sansone (European Bioinformatics Institute). Renzo Kottmann (Max Planck Institute for Marine Microbiology) followed this talk with an overview of the activities of the Genomic Standards Consortium in this domain. In particular, the talk reviewed GSC efforts to implement the "Minimum Information about a (Meta)Genome Sequence" (MIGS/MIMS) specification [5], in particular through the Genomic Contextual Data Markup Language (GCDML) [9]. Jeroen Raes (University of Brussels) then spoke on the need to standardize the outputs of basic computations as well and establish a MINImal MEtagenome Sequence analysis Standard (MINIMESS).

Norman Morrison (University of Manchester and the NERC Environmental Bioinformatics Centre) talked about progress made on the Environment Ontology and presented a new tool (Ontogrator) for integrating information from multiple databases through the markup of ontological terms, using examples from CAMERA, StrainInfo.net and The Genomes Online Database (GOLD). Finally, Folker Meyer (Argonne National Labs) gave an overview of the MG-RAST system, announcing that the next version of the system would implement the capture of MIMS for all metagenomic data sets.

### Session III. Meta-analysis

After lunch Iddo Friedberg (University of California San Diego and Miami University Ohio) chaired the third session of the day on Meta-analysis. Following on from Owen White's talk in the morning, Peer Bork (European Molecular Biology Laboratory) elaborated a vision for the European DACC and the International Human Microbiome Project (IHMP), showing early stage analyses of 16S data sets from key projects. Eric Alm (MIT) then presented an analysis of gene transfer between microbes using phylogenomic methods and discussed how this process could be dissected to understand the history of microbial evolution much like reading the geological record.

These two invited talks were followed by three contributed talks on projects designed to provide new ways of analyzing metagenomic data. These were from Daniel C. Richter (University of Tübingen) on the “Functional Classification of Environmental Reads using Gene Ontology”, Tom Matthews (Public Health Agency of Canada) on “Pathogen Profiling Pipeline: A metagenomics tool for rapid identification of pathogens from clinical specimens”, and Lucas A. Brouwers (Nijmegen Centre for Molecular Life Sciences) on “Pathway Signature Genes that are used to identify metabolic pathways in metagenomes”.

Following a coffee break that was combined with an afternoon poster session, Session III continued with three more invited talks. Maria Jesus Martin (European Bioinformatics Institute) described work at UniProt on GOS data in a talk entitled “Where is the metadata for downstream analyses?” and Jeffrey Grethe (UC San Diego) gave an update on the latest release of the CAMERA project which now makes extensive use of workflows and ontologies.

Eugene Kolker (Children's Hospital of Seattle) gave the last talk of the day on the "Premises and Promises" of 'omics during which he gave an overview of existing ‘omics, their success stories, limitations and challenges. This set the stage for the panel discussion that followed.

## Panel discussion

The meeting closed with a panel discussion on the Past, Present and Future of the GSC that was chaired by Dawn Field. The Panel included eight members of the GSC Board: Guy Cochrane, Lynette Hirschman, George Garrity, Eugene Kolker, Renzo Kottmann, Frank Oliver Glöckner, Susanna Sansone and Owen White. Dawn Field summarized the meeting and posed two questions. Harking back to the question posed by Renzo Kottmann in his presentation, “How should we develop the vision for the GSC” and “How should we work as a community to "minimize the mess," as Frank Oliver stated cleverly when introducing Jeroen Raes' talk on the MINIMESS proposal. She opened the discussion with questions from the audience.

Ben Temperton (PML) asked the Panel if it expected cloud computing to play an important role in the future of this community. This initiated a lively discussion about the potential need for science-specific computing clouds to handle the specific needs of the metagenomics community. In particular, Folker Meyer's presentation of the costs of basic bioinformatics analyses compared to future data generated by next-generation sequencing platforms spurred on the conversation, given that a 95 Gb Solexa run of the future could cost around $300k to analyze using blast on the Amazon cloud [10]. There was significant interest in whether the GSC could help the genomics and metagenomics community to speak 'with one voice' on this issue in order to work towards investment levels in a community-shared platform more akin to those built by the physics (e.g. colliders) and astronomy (radio telescopes) communities.

Folker Meyer and Owen White were designated to take this forward on behalf of the community and as a result the GSC subsequently formed a BioComputing Consortium. The Consortium is now working on a vision for a future "M5 platform" - adding both Models and MetaInfrastructure to the M3 concept. In brief, this group aims to bring together a set of strategic partners investing in various aspects of new and emerging technologies (workflows, grids, clouds, and turn-key desktop solutions [11]) to build a next-generation computing landscape. By working together as a community, the hope of this open membership group is to draw together a range of existing and future computational “jigsaw pieces” to create a new global platform. The architecture of this platform will be designed in direct response to the flood of data coming from next-generation sequencing technologies, in particular within the field of metagenomics. It will also be shaped by the understanding that the field of bioinformatics is rich in software. Fast, economical computing environments, including desktop solutions are now essential components of almost all research labs pursuing scientific questions using these data-rich technologies.

Peter Sterk (NERC Centre for Ecology and Hydrology) then asked the panel if a single global catalogue of metadata was still a high priority, especially given that MIGS/MIMS compliance was clearly increasing. He suggested that equally important was the capture and vetting of new terms for placement into relevant ontologies. The Panel re-affirmed the need for such infrastructure and discussions led by Renzo Kottmann (MPI-Bremen) and Peter Sterk (NERC CEH) following the meeting have formalized the proposal for a Phase II community-developed "Genomes and Metagenomes" (GEM) Catalogue based on the GSC's Genome Catalogue [12]. The GEM Catalogue will include GCDML [9] and the Genomic Rosetta Stone [13] at its core and will support a hub-and-spokes model of integrating several MIGS/MIMS compliant databases through web services. A full list of requirements can be found at the new project home page in the GSC wiki at http://gensc.org/. This set of requirements was further extended at the GSC 8 meeting to include intended support for multi-omic metadata capture and presentation through the use of the ISA infrastructure [14]. Finally, the concept of drawing a map of how all the standards communities work together and widening the scope to funders and journals was also discussed. Dawn Field suggested that a review of this landscape could be developed. This has subsequently fed into the concept of a Biosharing hub of information that is currently being built [15]. The aim of the BioSharing website (http://biosharing.org) is to help publicize and make transparent the activities that are ongoing in the community in the areas of standards, ontologies, tools, databases and policy development, encourage cross-talk among these community and foster increased engagement between researchers, technology providers, journals and funders.

## Conclusions

In summary, the M3 SIG meeting was very well attended. The "M3" concept worked well with many speakers combining all three aspects in their talks. Most notably, as a result of this workshop, the GSC has launched the M5 initiative and will explore holding similar meetings within the context of international society meetings. This already includes a follow-on GSC M3 workshop that will be held at the Pacific Symposium on Biocomputing (PSB) conference in Hawaii in January 2010.
